# Experimental Validation of the Sensitivity of Waveguide Grating Based Refractometric (Bio)sensors

**DOI:** 10.3390/bios5020187

**Published:** 2015-04-13

**Authors:** Thomas E. Gartmann, Florian Kehl

**Affiliations:** 1 CSEM Centre Suisse d’Electronique et de Microtechnique SA, Bahnhofstrasse 1, Landquart CH-7302, Switzerland; E-Mail: thomas.gartmann@csem.ch; 2 Laboratory of Biosensors and Bioelectronics (LBB), Swiss Federal Institute of Technology ETH, Gloriastrasse 35, Zürich CH-8092, Switzerland; 3 Optics Balzers AG, Neugrüt 35, Balzers FL-9496, Liechtenstein

**Keywords:** waveguide grating sensor, grating coupler, integrated optics, bulk refractive index sensitivity

## Abstract

Despite the fact that the theoretical foundations of the sensitivity of waveguide grating based (bio)sensors are well-known, understood and their implications anticipated by the scientific community since several decades, to our knowledge, no prior publication has experimentally confirmed waveguide sensitivity for multiple film thicknesses, wavelengths and polarization of the propagating light. In this paper, the bulk refractive index sensitivity *versus* waveguide thickness of said refractometric sensors is experimentally determined and compared with predictions based on established theory. The effective refractive indices and the corresponding sensitivity were determined via the sensors’ coupling angles at different cover refractive indices for transverse electric as well as transverse magnetic polarized illumination at various wavelengths in the visible and near-infrared. The theoretical sensitivity was calculated by solving the mode equation for a three layer waveguide.

## 1. Introduction

Waveguide grating based sensors are highly sensitive optical transducers, mainly applied for bulk refractometric or label-free (bio)sensing, to accurately determine the refractive index of a fluid or to detect the interaction, presence and concentration of (bio)molecules [1]. The application areas range from medicine, biotechnology and pharmaceutical industry to food, feed and environmental monitoring [2,3,4,5,6,7].

Evidently, a key parameter of such a sensor is its sensitivity. It is therefore important for the development of a new sensor to choose its overall design and the individual design parameters for a maximized sensitivity. Numerous different, highly sensitive planar waveguide sensor designs have been demonstrated (an overview can be found in [8,9,10]) and other publications focused on maximizing sensitivity and developing design rules for optimal sensors [1,10,11,12,13,14]. The aim of this publication is therefore neither to theoretically assess the sensitivity of said sensors nor to maximize it, but to provide experimentally measured data to verify well-established theory regarding the sensitivity of dielectric waveguide grating based (bio)chemical and refractometric sensors. These results have been anticipated for several decades but lack of a systematic experimental verification.

**Figure 1:**
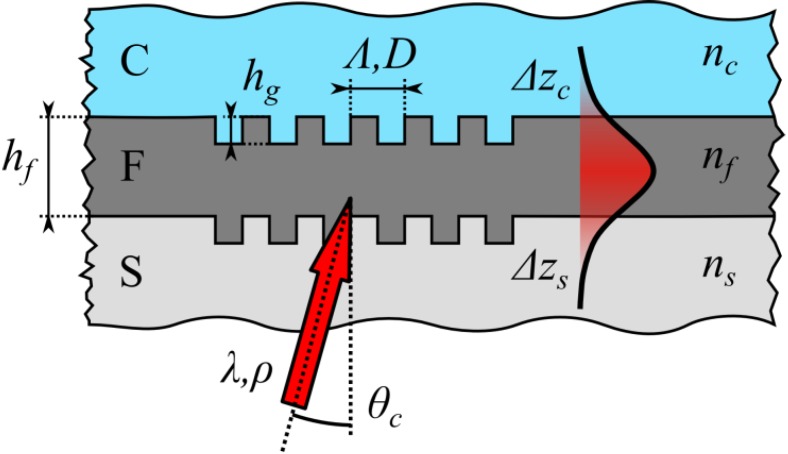
Schematic representation of a waveguide grating coupler, consisting of a substrate *S*, waveguide film *F* with a layer thickness of *h_f_* and cover layer *C* with refractive indices *n_s_*, *n_f_* and *n_c_*, respectively. A corrugated grating with a depth of *h_g_*, period *Λ* and duty-cycle *D* acts as a coupling element for coherent light with wavelength *λ*, polarization *ρ* incident at an angle *θ_c_*, thereby creating a guided mode with evanescent tails with penetration depths *Δz_c_* and *Δz_s_*.

In its simplest configuration, a planar, step-index waveguide grating coupler exhibits a 3-layer structure consisting of the supporting substrate *S*, a high refractive index waveguide layer *F* and the investigated cover layer *C* (Figure 1) [2,15,16]. A corrugated grating region in the waveguide acts both as a light coupling element into the waveguide by means of diffraction as well as the sensitive element of the sensor. The sensing principle of a grating coupler can be illustrated by the resonance condition for light coupling into or out of the waveguide via the grating [1,17]:

(1) $$n_{c/s}*\;sin\left(\theta_{c}\right)=n_{eff}-\frac{m_{g}\lambda }{\Lambda }$$

where *n_c/s_* denotes the refractive index of the cover or the substrate, depending from which side the sample is illuminated, *θ_c_* the coupling angle, *m_g_* the grating diffraction order, *λ* the vacuum wavelength of the incident light, *Λ* the grating period and

(2) $$n_{eff}=f(n_{c},\;n_{f},\;n_{s},h_{f},\;h_{g},\;D,\;\lambda ,\rho ,\;m)$$

the effective refractive index of the waveguide, which itself depends on the cover-, waveguide- and substrate refractive indices, the waveguide thickness *h_f_*, the depth *h_g_* and duty-cycle *D* of the corrugated grating, the wavelength *λ* and polarization *ρ* of the incident light, which can either be transverse electric (TE) or transverse magnetic (TM), as well as the mode number *m* of the propagating wave. Hereinafter, the influence of *h_g_* and *D* on *n_eff_* are neglected as only shallow and therefore weak gratings with *h_g_* << *λ* and two conformally corrugated waveguide sides with *D* ≈ 0.5 are considered [13].

As the coupling angle *θ_c_* depends on *n_eff_* (Equation (1)), which itself depends on the cover refractive index *n_c_* (Equation (2)), changes of the cover refractive index *n_c_* can be monitored by observing the in- or out-coupling angle [1]. As the sensing is accomplished by the evanescent tail of the propagating waveguide mode, the adsorption of molecules can be measured as the cover refractive index is altered in close proximity to the sensor surface, since in general, the adsorbed molecules exhibit a different refractive index than the displaced surrounding cover medium. Thus, a grating coupler can be employed as a (bio)chemical sensor [2]. To enhance the coupling capacity of chemical species to the sensor surface, hydrophilic and open hydrogel matrices with adlayer thicknesses *h_a_* of a few hundreds of nanometers are commonly anchored to the latter [18], hereby covering the entire extent of the evanescent field. In this common case, the sensitivity for surface sensing can be approximated by the sensitivity for homogeneous sensing, where the entire bulk refractive index of the cover changes. 

As a consequence of the above, the sensitivity *s* towards changes in the cover refractive index of said sensor can be expressed as the change of the effective refractive index with respect to the change of the bulk refractive index of the cover medium as defined in Equation (3).

(3) $$s=\;\frac{\partial n_{eff}}{\partial n_{c}}\approx \frac{\Delta n_{eff}}{\Delta n_{c}}$$

where *Δn_c_* denotes the difference in the refractive index of the cover material and *Δn_eff_* the resulting difference in the effective refractive index, which can be calculated with Equation (1) from experimentally determined coupling angles.

The sensitivity of a slab waveguide towards cover refractive index changes depends on the fraction of the total power *P* of the guided mode with respect to the power fraction in the cover *P_c_* [1]:

(4) $$\frac{P_{c/s}}{P}=\frac{n_{f}^{2}-n_{eff}^{2}}{n_{f}^{2}-n_{c}^{2}}\frac{\Delta z_{c/s}}{h_{eff}}$$

where *Δz_c/s_* are the penetration depths of the evanescent field into the cover and substrate layer, respectively, and *h_eff_* the effective waveguide thickness:

(5) $$h_{eff}=h_{f}+\Delta z_{c}+\Delta z_{s}$$

whereas the penetration depths depend on the polarization *ρ* of the propagating mode (*ρ* = 0 for TE and *ρ* = 1 for TM modes):

(6) $$\Delta z_{c/s}=\frac{\lambda }{2\pi }{\left(n_{eff}^{2}-n_{c/s}^{2}\right)}^{-\frac{1}{2}}{\left[{\left(\frac{n_{eff}}{n_{f}}\right)}^{2}+{\left(\frac{n_{eff}}{n_{c/s}}\right)}^{2}-1\right]}^{-\rho }$$

After some calculations (as further described in [1]) we can express the sensitivity toward cover refractive index changes as:

(7) $$s=\frac{\partial n_{eff}}{\partial n_{c}}=\left(\frac{n_{c}}{n_{eff}}\right)\left(\frac{P_{c}}{P}\right){\left[2{\left(\frac{n_{eff}}{n_{c}}\right)}^{2}-1\right]}^{\rho }$$

As aforementioned, the goal of this study is to experimentally validate and reconstruct the well-known, theoretical sensitivity plots *s(h_f_)* of waveguide grating couplers for various waveguide thicknesses *h_f_*, wavelengths *λ* and polarization *ρ* of the incident light. These can be obtained by numerically solving the transcendental three layer mode equation for *n_eff_* and inserting the obtained values in Equation (7), as explained in more detail in subchapter 2.2.

## 2. Material and Methods

### 2.1. Sample Preparation and Measurement of the Refractometric Sensitivity 

To determine the sensitivity of a given chip design, the in-coupling angles into the waveguide gratings were measured for different waveguide thicknesses *h_f_*, wavelengths *λ* and polarization *ρ* of the incident light as well as cover refractive indices *n_c_*. From the measured in-coupling angles, which were corrected with Snell’s law for the change in angle upon refraction at the transition from substrate to air, the effective refractive indices (Equation (1)) and the corresponding sensitivity *s* of the sensor towards change of the bulk refractive index were calculated (Equation (3)). In this study, only the case most often met in practice, where *n_c_* < *n_s_*, corresponding to an aqueous cover solution and a glass substrate, was considered.

**Figure 2:**
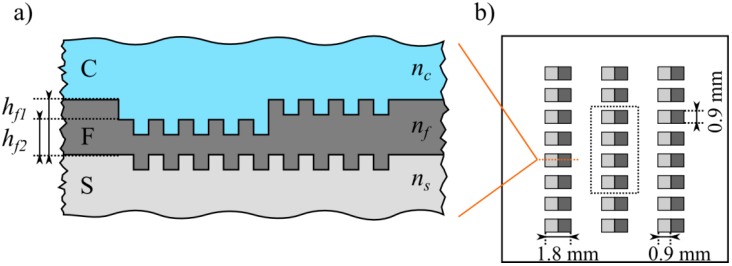
Schematic drawing of the investigated waveguide grating chips. (**a**) Cross section (not to scale); (**b**) Top view: Chip with 24 gratings with waveguide thickness *h_f_*_1_ and *h_f_*_2_, respectively, whereas the central eight gratings (four of each height) have been considered per measurement per chip.

**Figure 3:**
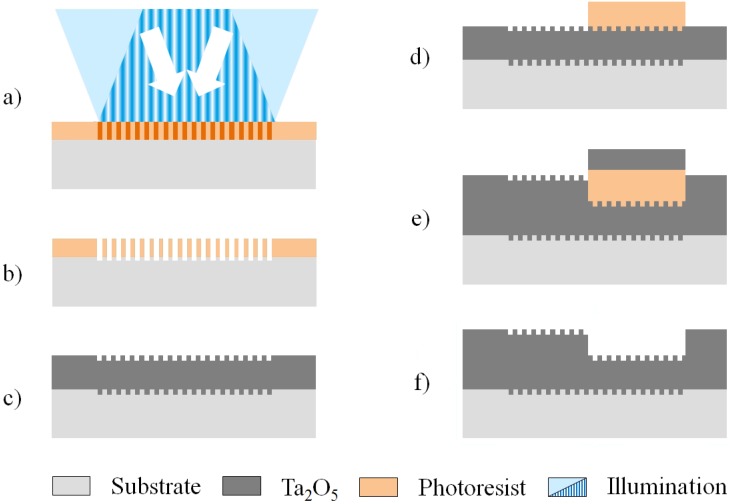
Chip production sequence: (**a**) exposure of the deposited photoresist to interference lithography; (**b**) photoresist development and subsequent etching of the substrate by reactive ion etching (RIE); (**c**) O_2_ plasma stripping of the photoresist and sputtering of a first Ta_2_O_5_ layer; (**d**) deposition and structuring of a sacrificial photoresist; (**e**) sputtering of a second Ta_2_O_5_ layer and (**f**) lift-off of the additional Ta_2_O_5_ by removing the sacrificial photoresist.

The waveguide of the investigated sensor consists of a Ta_2_O_5_ film on a structured glass substrate (D263T by Schott, Mainz, Germany), as schematically depicted in Figure 2. To facilitate coupling via the substrate, a broadband anti-reflective coating was deposited on its reverse side. In a second production step, a rectangular grating (0.9 mm by 0.9 mm) was structured into the glass substrate using interference photolithography and reactive ion etching (RIE) in a CHF_3_/Ar plasma (Figure 3). The developed photoresist was removed by O_2_ plasma stripping and subsequently, a first layer of Ta_2_O_5_ was magnetron sputtered onto the substrate, followed by the deposition of a sacrificial photoresist layer in the regions where a thinner waveguide thickness was desired. A second layer of Ta_2_O_5_ was sputtered onto the sample thereafter and the production was completed with a lift-off process to uncover the thinner waveguide regions. Average waveguide thicknesses *h_f_* ranging from 83.0 nm ± 0.6 nm to 329.63 nm ± 0.08 nm with a root mean square (RMS) surface roughness of approximately 1.2 nm were produced. The waveguide thicknesses were measured using a spectrometer (Lambda 800, PerkinElmer, Waltham, MA, USA), as well as with a prism coupler (Model 2010, METRICON, Pennington, NJ, USA). The rectangular gratings were produced with a grating depth *h_g_* of 12 ± 2 nm and a grating period *Λ* of 360 ± 0.1 nm.

**Figure 4:**
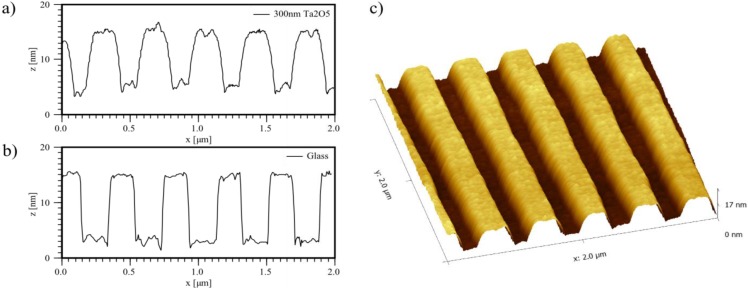
By atomic force microscopy (AFM) measured profile of the grating after (**a**) and before (**b**) deposition of ~300 nm Ta_2_O_5_, together with a topographical scan of the grating (**c**) after deposition. Due to the small aspect ratio of the grating (*h_g_* << *Λ*), the structure of the grating is mostly conserved also for thicker Ta_2_O_5_ layers.

Several samples were examined by atomic force microscopy (AFM) to measure the RMS surface roughness as well as to ensure the envisaged grating structure and confirm the conformity of the two corrugated interfaces *S-F* and *F-C*, which was inherently granted due to the grating’s small aspect ratio (*h_g_* << *Λ*) (Figure 4). This particular chip design with two different waveguide thicknesses is based on the WIOS sensor [19,20], a standard product at Optics Balzers, with the advantage of the production process being readily available, stable and well understood. Whereas the configuration with two adjacent waveguide regions with two different thicknesses are a prerequisite for the WIOS sensor, it was not a requirement for the measurements conducted in the framework of this study, but still beneficial, as two different thicknesses could be investigated at once.

**Figure 5:**
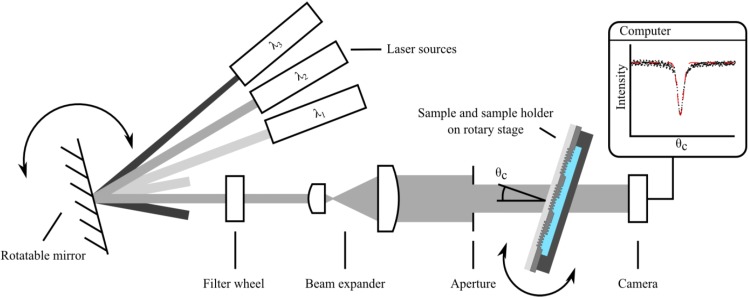
Experimental setup to determine the coupling angles consisting of various laser sources, rotatable mirror for source selection, filter wheel with polarizers for TE and TM polarization selection, beam expander, aperture, the mounted sample on a motorized rotation stage as well as a CCD camera for signal recording.

**Figure 6:**
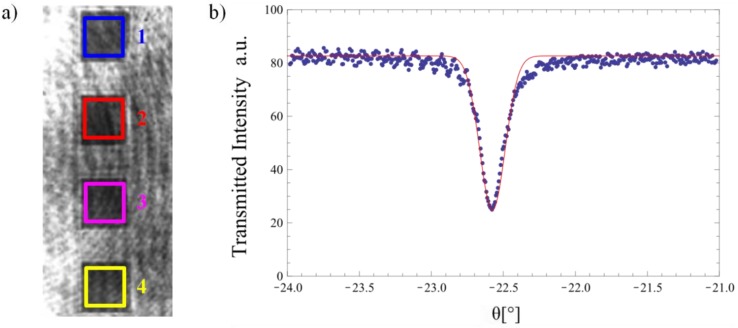
Typical measurement of the transmitted light intensity at the coupling angle: (**a**) CCD camera image with the four measurement regions. (**b**) Measured intensity and inverted Gaussian fit for one region to determine the coupling angle. The intensity oscillations are caused by Fresnel reflections at cover and substrate. Configuration: *λ* = 532.3 ± 0.2 nm, *h_f_* = 122.8 ± 0.8 nm, *n_c_* = 1.5247.

The experimental setup, similar to the one featured in [21] to study final grating length effects, is sketched in Figure 5. The sensor chips were mounted in a transparent PMMA sample holder and different cover media were applied to the corrugated Ta_2_O_5_ surface. The investigated media included air, purified water (Milli-Q^TM^, EMD Millipore, Billerica, MA, USA) and index matching liquid (Series A, *n* = 1.52, Cargille Laboratories, Cedar Grove, NJ, USA). The mounted samples were placed on a motorized rotary stage with encoder (CR1/M-Z7, Thorlabs, Newton, NJ, USA) with an angular repeatability of less than 0.017 degree and an angular resolution of 6·10^−4^ degree. Afterwards, the samples were illuminated through the substrate with linearly polarized light at wavelengths of 532.3 ± 0.2 nm (CW532, Roithner LaserTechnik, Vienna, Austria), 632.8 ± 0.2 nm (1103P, Uniphase, Mateca, CA, USA), 779.7 ± 0.2 nm (LDM780/3LJ, Roithner LaserTechnik, Vienna, Austria) and 845.1 ± 0.2 nm (LDM850/3LJ, Roithner LaserTechnik, Vienna, Austria), according to the available laser sources within the investigated wavelength range. The emission spectra of the laser sources were previously measured with an optical spectrum analyzer (AQ6373, Yokogawa, Musashino, Japan). To determine the in-coupling angle, the angle dependent light transmission through the grating region was measured with a CCD camera combined with a telecentric lens (Guppy F-033B by Allied Vision Technologies, Stadtroda, Germany and 0.5× TML 63074 by Edmund Optics, Barrington, NJ, USA). For every combination of waveguide thickness, cover refractive index as well as polarization and wavelength of the incident light, 4 out of 24 individual grating regions per chip (Figure 2b and Figure 6a) were measured in parallel to allow for an accuracy estimate of the determined coupling angles. The angle of incidence was swept by turning the mounted sample on the rotary stage, which was controlled via a MATLAB [22] script from an external computer. At the in-coupling angle, the intensity of the transmitted light was decreased as a part of the incident light was coupled into the waveguide, resulting in a dip in the measured transmitted light intensity *versus* angle.

A typical example of such a measurement is shown in Figure 6. Subsequently and in good approximation to the curve, a Gaussian fit was applied to the inverse of the dip and the center of the fit was defined as the in-coupling angle [21]. Since coupling into the waveguide occurs symmetrically around the angle of normal light incidence onto the waveguide (see Equation (1)), measurements were performed while turning the rotary stage clockwise (+) as well as counter-clockwise (−) from the angle of normal incidence of the light onto the sample. By evaluating the difference between the resulting positive and negative coupling angles, it was thus possible to precisely calculate the angle of normal light incidence onto the sample and therefore to correct the measured in-coupling angles for an offset.

From the measured in-coupling angles, the effective refractive indices were calculated with Equation (1) and subsequently the sensitivity with Equation (3) for two different cases; in the first case, the difference Δ*n_c1_* between air and water and in the second the difference Δ*n_c_*_2_ between water and the index matching liquid was evaluated. These measurements were performed with TE and TM polarization of the incident light. Furthermore, only the first two diffraction orders *m*_g_ = ±1 of the grating and excitation of the waveguide’s fundamental mode was investigated, according to the coupling condition in Equation (1).

### 2.2. Calculation of the Theoretical Sensitivity

The theoretical sensitivity was calculated by numerically solving the transcendental three-layer mode equation (Equation (8)) to compute *n_eff_*

(8a) $$\frac{2\pi }{\lambda }\sqrt{n_{f}^{2}-n_{eff}^{2}}\;h_{f}+\Phi_{c}+\Phi_{s}-m\pi =0$$

where

(8b) $$\Phi_{c/s}=-tan^{-1}\left[{\left(\frac{n_{f}}{n_{c/s}}\right)}^{2\rho }\frac{\sqrt{n_{eff}^{2}-n_{c/s}^{2}}}{\sqrt{n_{f}^{2}-n_{eff}^{2}}}\right]$$

and *m* = 0 for the considered fundamental modes. The measured wavelengths of the incident light were directly fed into Equation (8) along with the corresponding refractive indices listed in Table 1. The refractive indices of the liquids were either provided by the manufacturer (for the index matching liquid) or by literature (for water [23]). The refractive index of air was set to *n_air_* = 1.0003 for all investigated wavelengths [24], whereas the refractive indices of the substrate and the Ta_2_O_5_ film were determined with the aforementioned prism coupler. With these input parameters the effective refractive indices *n_eff_* were calculated for the three different cover refractive indices. Together with the cover refractive index difference, the corresponding sensitivity was calculated with Equation (3).

**Table 1:** Refractive Indices of the Sensor Materials at the Investigated Wavelengths.

*λ* [nm]	*n_f_*	*n_s_*	*n_water_*	*n_index·matching·liquid_*
532.3	2.1511	1.5264	1.3354	1.5247
632.8	2.1229	1.5213	1.3321	1.5173
779.7	2.1024	1.5168	1.3290	1.5115
845.1	2.0918	1.5157	1.3279	1.5099

## 3. Results and Discussion

**Figure 7:**
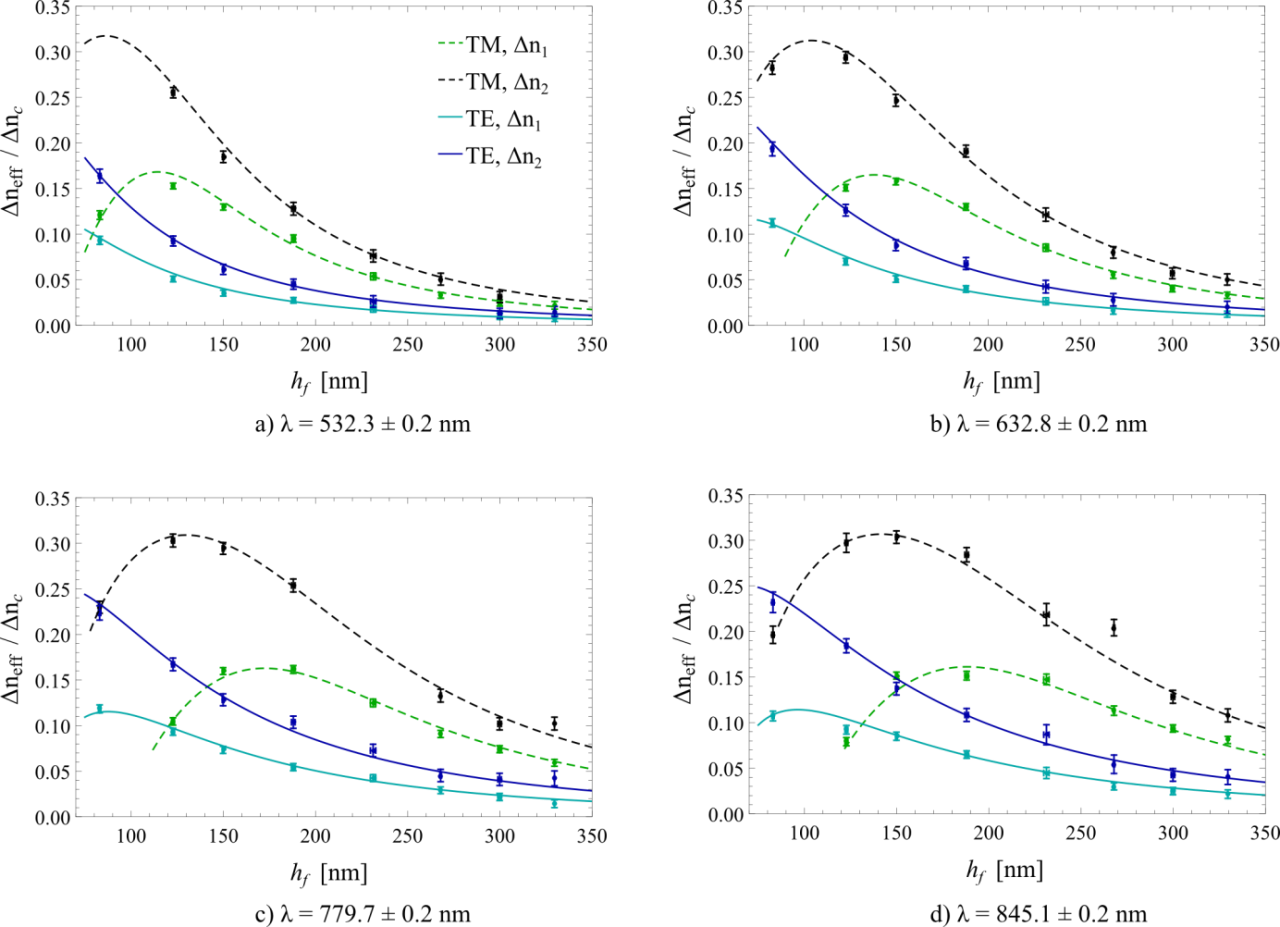
Measured (marks) and calculated sensitivities (lines) for transverse magnetic (TM) and transverse electric (TE) polarization at the four investigated wavelengths of the incident light. All error bars (standard deviations of all measured values) were plotted but some are barely discernible due to their small value.

Both measured and calculated sensitivities for the investigated waveguide grating based (bio)sensor are displayed in Figure 7 for all measured waveguide thicknesses, wavelengths and polarizations of the incident light. There is a good agreement between the measured data and the numerical simulations. To quantitatively express the agreement, the root-mean-square deviation (RMSD) of the measured sensitivities from the simulated ones was calculated for every displayed graph in Figure 7. The RMSD represents the deviation of the simulated values from the measured ones, or vice versa*.* On average, the RMSD was 0.006 ± 0.003, which is equal to the average standard deviation of the measured sensitivities. Thus, the accuracy is most probably limited by the achievable resolution of the current measurement setup. Additionally, the Pearson product-moment correlation coefficients (PPMCC) were calculated for all the graphs in Figure 7. The PPMCC were ≥0.994, except for *λ* = 845.1 ± 0.2 nm and TM polarization with PPMCCs of 0.973 (Δ*n_c_*_1_) and 0.984 (Δ*n_c_*_2_), signifying that the measured and simulated sensitivities are almost perfectly correlated. Therefore, it can be concluded, that the good agreement of measurements and simulations is supported by the calculated RMSD’s and PPMCC’s.

Regarding the measured sensitivity values, one can observe that for TE modes, highest sensitivity is achieved in close proximity to the cut-off thickness of the fundamental mode. By contrast, for TM polarization, waveguide layer thicknesses further away from the cut-off in the range of 100 nm to 170 nm exhibit highest sensitivity towards cover refractive index changes for the investigated range of refractive indices, wavelengths and grating structures. In general, it can be concluded that for homogeneous sensing, the fundamental TM mode exhibits a higher sensitivity over the corresponding TE mode for the investigated case where *n_c_* < *n_s_*. This can easily be concluded from Equation (7) and is in agreement with the literature [1,11,14,25]. For both polarizations, a general trend of decreasing sensitivity for increasing waveguide thicknesses can be observed. By considering Equations (4) and (5), this can be explained by the power fraction of the mode overlap of the propagating light protruding into the cover medium, which is inversely proportional to the effective waveguide thickness *h_eff_*. Therefore, with increasing *h_eff_*, the sensitivity approaches zero as *P_c_/P*→0 [1]. In addition, one can observe that for high refractive indices of the cover materials, in this work the measurement of water-index matching liquid, the sensitivity is increased compared to using cover media with lower refractive indices. This is a direct consequence governed by Equation (6), as the evanescent field in the cover medium *Δz_c_* tends towards infinity as *n_eff_* → *n_c_*, resulting in a maximized fraction of total power in the cover medium (*P_c_/P*→1).

As mentioned in the introduction, the aforesaid findings only hold true for homogeneous, refractometric sensing and for biosensing with 3D immobilization matrices with thicknesses in the range of or bigger than the evanescent field’s penetration depth (*h_a_* ≥ *Δz_c_*). The case of surface or thin-layer sensing (*h_a_* << *Δz_c_*) has been investigated theoretically as well as experimentally in [1,11,14,26], although the conditions for maximum sensitivity are close to the homogeneous case. A normalized analysis for the sensitivity optimization of waveguide based sensors can be found in [14].

It should also be mentioned that the investigated cover refractive index changes are substantially bigger than in typical sensing applications, where the effect of adsorbing biomolecules on the cover refractive index is several orders of magnitude smaller. Nevertheless, this does not contradict the abovementioned calculations. If all parameters are known (which is the case here), the effective refractive index *n_eff_* is unambiguously defined via Equation (2). For the calculation and measurement of *n_eff_* with one cover medium, no parameters of the second cover medium are required. Therefore, these calculations are decoupled and the magnitude of Δ*n_c_* has no influence on the accuracy of the determination of the effective refractive indices and the derived sensitivity (Equation (3)).

## 4. Conclusions

In this publication, the refractometric sensitivity of waveguide grating sensors was experimentally determined for different waveguide thicknesses, wavelengths and polarizations of the incident light and compared with numerical calculations to verify well-established theory.

A good agreement between theoretically calculated and experimentally measured sensitivity was observed. The RMSD’s of the measured values from the simulated values are in the same order of magnitude as the experimental uncertainty of the measured sensitivities. Further, a very good correlation of the measured and simulated sensitivities was observed, yielding PPMCC’s above 0.97. Therefore, it can be concluded that the sensitivity of the coupling angle towards change of the bulk refractive index can be accurately and reliably modeled with established theory. Hence, this study aims at filling a gap in the published literature by experimentally reconstructing the sensitivity curves for waveguide grating coupler based sensors and it confirms the validity as well as the accuracy of the theoretical predictions for various illumination wavelengths, polarizations, waveguide thicknesses and refractive indices of the cover medium.
